# Intraosseous microdialysis for bone free flap monitoring in head and neck reconstructive surgery: A prospective pilot study

**DOI:** 10.1002/micr.30529

**Published:** 2019-10-22

**Authors:** Stéphanie Dakpé, Emilien Colin, Jérémie Bettoni, Julien Davrou, Momar Diouf, Bernard Devauchelle, Sylvie Testelin

**Affiliations:** ^1^ Department of Maxillofacial Surgery and Stomatology Amiens‐Picardie University Hospital Amiens France; ^2^ EA 7516 CHIMERE Université de Picardie Jules Verne Amiens France; ^3^ Facing Faces Institute Amiens‐Picardie University Hospital Amiens France; ^4^ Department of Maxillofacial Surgery and Stomatology La Pitié‐Salpêtrière University Hospital, AP‐HP Paris France; ^5^ Clinical Research Department Amiens‐Picardie University Hospital Amiens France

## Abstract

**Background:**

Although some researchers have positioned microdialysis catheters in the soft tissue surrounding bone, the results did not accurately reflect bone metabolism. The present study's objective was to establish the feasibility of microdialysis with a catheter positioned directly in bone.

**Methods:**

Thirty‐four patients (19 males, 15 females; median age: 59) were included in a prospective, nonrandomized clinical trial in the Department of Maxillofacial Surgery at Amiens‐Picardie University Hospital (Amiens, France). Fibula or iliac crest free flaps were used in reconstructive head and neck surgery (for cancer, osteoradionecrosis, trauma, or ameloblastoma) and monitored with microdialysis catheters positioned in a hole drilled into the bone. Glucose, lactate, pyruvate, and glycerol concentrations were analyzed for 5 days.

**Results:**

All catheters were positioned successfully, and thrombosis did not occur during the monitoring. In two patients, an increase in the lactate concentration and a glucose level close to 0 were associated with signs of flap necrosis, with removal on Days 9 and 50. In viable flaps, the mean glucose level was 2.02 mmol/L, the mean lactate level was 8.36 mmol/L, and the mean lactate/pyruvate ratio was 53. Forty percent of the glucose values were below 1 mmol/L, and 50% of the lactate/pyruvate ratio values were above 50—suggesting a specific metabolic pattern because these values would be considered as alert values in soft tissue.

**Conclusion:**

Monitoring bone free flaps with intraosseous microdialysis is feasible. This technique specifically assesses bone viability, and further studies are now necessary to define the alert values in bone.

## 1 INTRODUCTION

Most studies of free flap monitoring (including all types of free flaps and reconstructive surgery sites, and various monitoring techniques) have found similar success rates (97–99%) and salvage rates (up to 80%) (Chae et al., 2015). Nevertheless, there is no consensus in the literature on the optimal algorithm for the intraoperative assessment of tissue perfusion (Smit et al., 2018). Monitoring is even more difficult for buried bone flaps, and the standard evaluation (currently a clinical assessment based on the color, capillary refill, and bleeding pattern) is not satisfactory. The failure rate in reconstructive surgery with bone free flaps (11–25%, depending on the study) is higher than that with muscle, fasciocutaneous, or cutaneous flaps (1–17%) (Benacquista, Kasabian, & Karp, 1996; Bui et al., 2007; Ferguson Jr & Yu, 2009; Jallali, Ridha, & Butler, 2005). Over the past 15 years, several monitoring methods for head and neck reconstructive surgery have therefore been developed: implantable Doppler, color duplex sonography, near‐infrared spectroscopy, laser Doppler flowmetry, and microdialysis (Abdel‐Galil & Mitchell, 2009; Hölzle et al., 2010; Kääriäinen, Halme, & Laranne, 2018). At present, many surgeons consider that microdialysis (initially developed by Delgado, DeFeudis, Roth, Ryugo, and Mitruka (1972)) is a reliable monitoring method for buried free flaps and can detect flap compromise early (Brix, Muret, Ricbourg, & Humbert, 2006; Frost et al., 2015; Jyränki, Suominen, Vuola, & Bäck, 2006; Nielsen, Gutberg, & Birke‐Sørensen, 2011). Microdialysis provides an accurate, objective measurement of the flap's metabolism during ischemia, and thus produces an early, sensitive indication of flap failure (Chae et al., 2015).

The concept of microdialysis is based on the sampling of metabolites present in the interstitial liquid (glucose, lactate, pyruvate, and glycerol) and whose concentrations vary in the event of tissue hypoxia (Delgado et al., 1972). When perfusion is impaired (e.g., by ischemia), the glucose and pyruvate levels decrease and the lactate level increases as a result of anaerobic metabolism. These changes are ultimately followed by an increase in glycerol levels related to cell lysis.

Ungerstedt and Rostami (2004) established the usual normal values for soft tissue based on the studies of the brain: 2 mmol/L for glucose, 120 μmol/L for pyruvate, 2 mmol/L for lactate, and 15 to 20 for the lactate/pyruvate ratio. The researchers further stated that the lactate/pyruvate ratio appears to be a reliable marker of tissue ischemia since there is a statistically significant correlation between the lactate/pyruvate ratio and clinical outcomes. Hence, Ungerstedt and Rostami defined a lactate/pyruvate ratio >25 as a warning sign for metabolic crisis and energy deficiency. In the same year, Setala et al.'s study of a microvascular flap model showed that a decrease in the glucose concentration and an increase in the lactate concentration were associated with arterial and venous occlusion (Setälä et al., 2004). Some years later, Birke‐Sørensen, Toft, and Bengaard (2010) specified the alert values in the case of soft tissues transfer. Compromise of a muscle‐free flap can be suspected when the glucose level falls below 1 mmol/L, when the lactate level exceeds 15 mmol/L, and when the lactate/pyruvate ratio increases above 25. Although a few researchers have used microdialysis to monitor bone free flaps (Laure, Sury, Bayol, & Goga, 2009; Mourouzis, Anand, Bowden, & Brennan, 2007), they all placed the catheter in the surrounding muscle or in a skin paddle; these sites do not, however, reflect bone vitality. Only Bøgehøj, Emmeluth and Overgaard (2007) used intraosseous microdialysis to evidence ischemia in human femoral heads removed during total hip replacement. Nevertheless, an accurate assessment of bone vascularization is essential for improving bone free flap survival rates. We hypothesized that a catheter placed directly into a bone free flap can assess local perfusion by monitoring glucose, lactate, and pyruvate levels and lactate/pyruvate ratio. To test this hypothesis, we conducted a pilot clinical study of the feasibility and reliability of intraosseous microdialysis for bone free flap monitoring in head and neck reconstructive surgery.

## 2 PATIENTS AND METHODS

This prospective, single‐center, nonrandomized, pilot clinical study (http://clinicaltrials.gov identifier: NCT01879384) was conducted in the Department of Maxillofacial Surgery at Amiens‐Picardie University Medical Center (Amiens, France). The study was approved by the local institutional review board (*CPP Nord‐Ouest II*: reference 2010/42, ID‐RCB 2010‐A01176‐33). All participants provided their written, informed consent prior to inclusion. The study was conducted in accordance with the tenets of the 1975 Declaration of Helsinki and its subsequent amendments.

Each patient due to undergo reconstructive facial surgery involving a bone free flap was invited to participate in the study. The main inclusion criteria were aged over 18, an indication for head and neck reconstructive surgery with an iliac crest or fibula free flap, and the provision of informed consent. The main exclusion criteria were aged under 18, and reconstructive surgery with another type of flap.

The cohort comprised 34 adult patients (19 males and 15 females) who underwent primary or secondary facial reconstruction with either an iliac crest free flap (*n* = 13, 38.24%) or a fibula free flap (*n* = 21, 61.76%) (Table 1). The indications for surgery were variously head and neck cancer (*n* = 13, 38.24%), osteoradionecrosis (*n* = 12, 35.29%), trauma (*n* = 6, 17.65%), and ameloblastoma (*n* = 3, 8.82%). The median (range) age was 59 (18–70), and 76.47% (*n* = 26) of the patients were smokers. The arterial anastomoses were end‐to‐end in 97.06% of the cases (*n* = 33), and the venous anastomoses were end‐to‐end in 76.47% (*n* = 26). The receiving vessel was the superior thyroid artery in 29.41% of the cases (*n* = 10), the facial artery in 38.24% (*n* = 13), the external carotid artery in 20.59% (*n* = 7), and the lingual artery in 11.76% (*n* = 4). The venous connection was the thyrolinguofacial trunk in 44.12% of the cases (*n* = 15), the external jugular vein and the facial vein in 20.59% each (*n* = 7 each), and the internal jugular vein in 14.71% (*n* = 5). The mean ± *SD* duration of ischemia was 90.48 ± 35.15 min (Table 2).

**Table 1:** Characteristics of the study population

Variable		Population (*n* = 34)
Age, years, median (range)		59 (18–70)
Gender	Male, *n* (%)	19 (55.88)
	Female, *n* (%)	15 (44.12)
Tobacco consumption, *n* (%)		26 (76.47)
Alcohol consumption, *n* (%)		13 (38.24)
High blood pressure, *n* (%)		8 (23.53)
Arteriosclerosis, *n* (%)		5 (14.70)
Diabetes mellitus, *n* (%)		4 (11.76)
Radiotherapy or chemotherapy, *n* (%)		17 (50)
Indication	Head and neck cancer, *n* (%)	13 (38.24)
	Osteoradionecrosis, *n* (%)	12 (35.29)
	Trauma, *n* (%)	6 (17.65)
	Ameloblastoma, *n* (%)	3 (8.82)

**Table 2:** Characteristics of the surgical procedures

Variable		Surgical procedures (*n* = 34)
Flap type	Iliac crest, *n* (%)	13 (38.24)
	Fibula, *n* (%)	21 (61.76)
Ischemia duration, min, mean ± *SD*		90.48 ± 35.15
Recipient artery	Facial, *n* (%)	13 (38.24)
	Superior thyroid, *n* (%)	10 (29.41)
	External carotid, *n* (%)	7 (20.59)
	Lingual, *n* (%)	4 (11.76)
Type of anastomosis on recipient artery	End‐to‐end, *n* (%)	33 (97.06)
	End‐to‐side, *n* (%)	1 (2.94)
Recipient vein	Thyrolinguofacial trunk, *n* (%)	15 (44.12)
	Facial, *n* (%)	7 (20.59)
	External jugular, *n* (%)	7 (20.59)
	Internal jugular, *n* (%)	5 (14.71)
Type of anastomosis on recipient vein	End‐to‐end, *n* (%)	26 (76.47)
	End‐to‐side, *n* (%)	8 (23.53)

### 2.1 Microdialysis equipment

A microdialysis catheter (CMA 70®; membrane cut‐off: 20 kDa, shaft length: 130 mm; diameter: 0.9 mm; CMA Microdialysis AB, Stockholm, Sweden) was linked to a syringe (CMA 106/107 Pump Syringe®; CMA Microdialysis AB) containing a solution for perfusion (Perfusion fluid T1®, containing Na^+^ (147 mmol/L), K^+^ (4 mmol/L), Ca^2+^ (2.3 mmol/L) and Cl^−^ (156 mmol/L); CMA Microdialysis AB), and it was driven by a pump (CMA 106 Microdialysis Pump®; CMA Microdialysis AB). Each pump was powered by a separate battery (1 × 3 V battery for the CMA 106/107 Pump®; CMA Microdialysis AB), and the circuit was perfused at a fixed flow rate of 0.3 μL/min. Microdialysis samples were collected in microvials (CMA Microdialysis AB) placed in the vial holder at the end of the catheter.

### 2.2 Intervention and surgical procedure

All patients underwent facial reconstructive surgery using either an iliac crest free flap or a fibula free flap. In addition to routine clinical monitoring, bone free flaps were monitored using the microdialysis catheter positioned directly in the bone tissue. Flap harvesting and shaping (including osteosynthesis) and surgery were performed according to standard techniques. In the cases of cancer resection, the neck was dissected. Microsurgical anastomoses were end‐to‐end or side‐to‐end. Before closure, a hole (diameter: 2.3 mm) was drilled into the bone part of the flap. A sterile, double lumen microdialysis catheter was placed through the skin into the bone using a splittable introducer and was anchored to the skin with a suture stitch. The microdialysis pump and perfusion solution were connected immediately after implantation of the catheter. The dialysate was monitored for 5 days; the samples were analyzed every hour on Day 0, every 2 hr on Day 1, and every 3 hr on the following days. The dialysis fluid collected in the microvials was analyzed using an ISCUSflex® analyzer (CMA Microdialysis AB), with an automatic, real‐time graphical display for the level of each metabolite (including glucose, lactate, pyruvate, and glycerol). The data were processed using LABpilot® software (CMA Microdialysis AB). At the end of the monitoring period, the catheter was easily removed.

### 2.3 Data analysis

Quantitative variables were expressed as the mean ± *SD*, the mean ± *SE* of the mean, and the mean [90%CI] or the median (range). Qualitative variables were expressed as the number (percentage). Distributions of quantitative variables were displayed as histograms. For patients without flap compromise, the mean [90% confidence interval (CI)] was calculated using a random‐effect analysis of variance for repeated measures. The mean [90%CI] for time curves was calculated from the mean and the standard error of the mean at each sampling time.

All statistical analyses were performed using the SAS® software (version 9.2, SAS Institute, Cary, NC), and the results were represented graphically using the RStudio software (version 1.0.143, http://www.rstudio.com) for the R statistical computing environment (http://www.r-project.org/).

The analyzed patient‐related variables were age, sex, and medical history (Table 1), and the analyzed intervention‐related variables included flap type, duration of ischemia, and recipient vessels used for microsurgical anastomosis (Table 2). The outcome of the flap (compromised or not comprised) was assessed 3 months after surgery.

Technical feasibility was based on the following criteria: the immediate success rate of catheter positioning, the proportion of catheters still in place 5 days after placement, the number of metabolite values (glucose, lactate, pyruvate, and glycerol) obtained during monitoring, the compliance rate of the team collecting the microvials (the number of microvials actually collected divided by the expected number of microvials over the 5 days [*n* = 60]).

**Figure 1:**
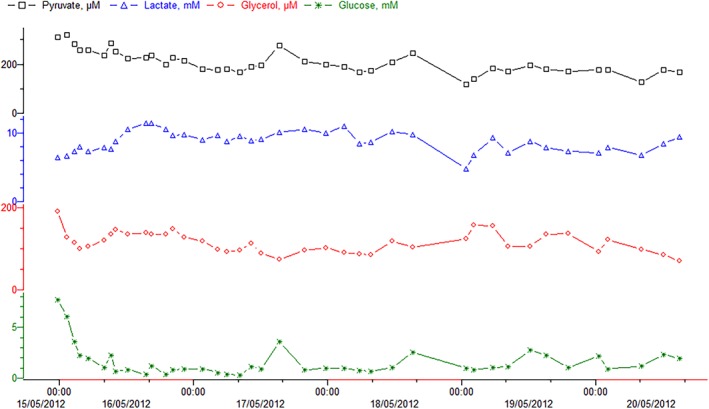
Time course curves for metabolites in viable flaps. Glucose is shown in green, with lactate in blue, glycerol in red, and pyruvate in black. The equilibration period was determined retrospectively

The mean levels and kinetic parameters were calculated for the glucose and lactate concentrations and the lactate/pyruvate ratio. Finally, the equilibration period (defined as the time required for the achievement of stable metabolite concentrations, as monitored by microdialysis) was estimated for each patient by the retrospective observation of the time curves (Figure 1).

## 3 RESULTS

The catheter was successfully positioned in all cases, and 88.2% (*n* = 30) of the catheters were in place after 5 days. Three patients removed accidently their microdialysis catheter on Day 2, and one patient removed it on Day 3. The mean duration of microdialysis monitoring was 4.04 ± 1.22 days. The nursing staff collected 1,410 microvials, corresponding to a compliance rate of 80.39%.

**Figure 2:**
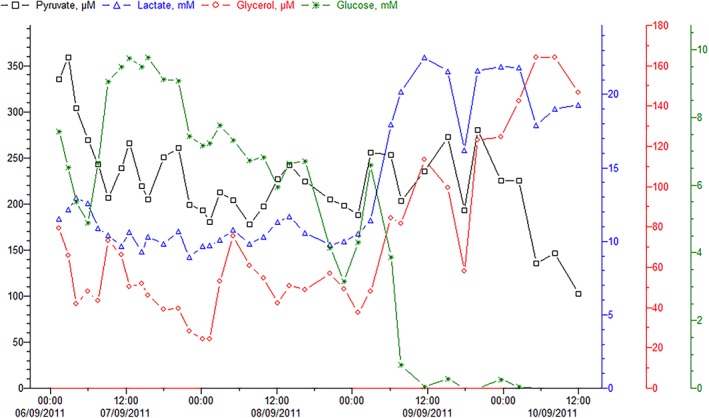
Time course curves for metabolites in the two ischemic flaps. Glucose is shown in green, with lactate in blue, glycerol in red, and pyruvate in black

**Figure 3:**
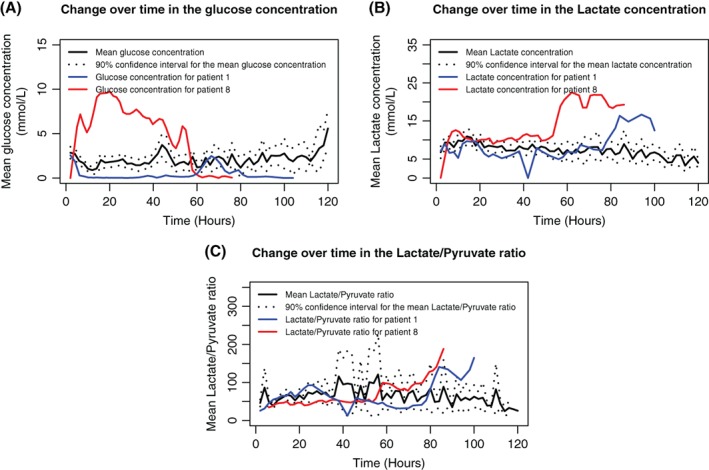
Change over time in the glucose (A) and lactate (B) concentrations and in the lactate/pyruvate ratio (C) for the two ischemic flaps versus the mean values for the viable flaps

**Figure 4:**
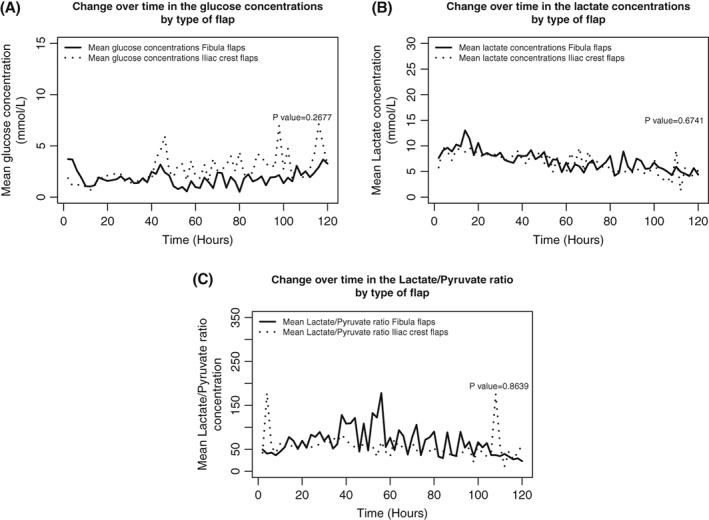
Change over time in the glucose (A) and lactate (B) concentrations and in the lactate/pyruvate ratio (C), by type of flap (fibula flaps and iliac crest flaps)

**Figure 5:**
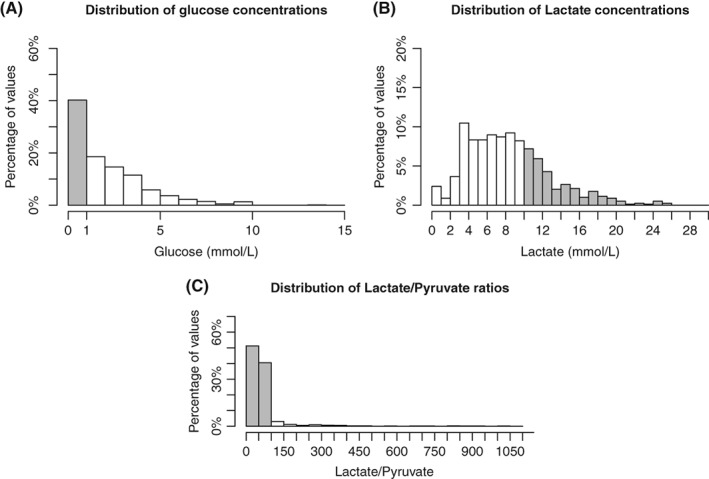
Distribution of glucose (A) and lactate (B) concentrations and of lactate/pyruvate ratio (C) in viable flaps

In a clinical assessment, none of these flaps was found to be compromised during the first 5 days of follow‐up. Nevertheless, an increase in the lactate concentration (by 146% and 224%, respectively) and a glucose concentration close to 0 mmol/L were noted in Patients 1 (Table 3 and Figure 3) and 8 (Table 3, Figure 2 and Figure 3); these features were later associated with clinical signs of flap necrosis, such as delayed wound healing, leakage, and typical odor. During flap revision, evidence of pedicle thrombosis and the absence of bleeding after bone drilling prompted us to remove the flaps on Days 9 and 50, respectively. The outcome at Month 3 was satisfactory for 94.12% of the flaps (*n* = 32). The mean equilibration period was determined to be 5.05 ± 1.65 hr. For patients without flap compromise, the mean [90%CI] glucose level was 2.02 mmol/L [0–5.50], the mean lactate level was 8.36 mmol/L [1.60–15.13], and the mean lactate/pyruvate ratio was 53 [8–99]. The mean changes over time in the glucose level, lactate level, and lactate/pyruvate ratio are presented in Table 3 and Figure 3A–C, respectively. The mean glucose and lactate concentrations and the mean lactate/pyruvate ratio did not differ significantly as a function of gender (*p* = .097, *p* = .543, and *p* = .922, respectively) or the type of flap (*p* = .268, *p* = .674 and *p* = .864, respectively) (Figure 4A–C). The distributions of the glucose and lactate levels and the lactate/pyruvate ratio are shown in Figure 5A–C, respectively. Forty percent of the glucose values measured during the monitoring period were below 1 mmol/L, and 50% of the lactate/pyruvate ratio values were above 50 (Figure 5).

**Table 3:** Microdialysis readings (glucose and lactate concentrations, and the lactate/pyruvate ratio) for the two ischemic flaps versus the mean values for the viable flaps

Sample #	Glucose (mmol/L)			Lactate (mmol/L)			*L*/*P* ratio		
	Mean	Patient 1	Patient 8	Mean	Patient 1	Patient 8	Mean	Patient 1	Patient 8
1	2.85	2.57	0.00	6.77	7.14	0.05	46.11	25.79	NA
2	2.74	1.95	NA	9.33	8.97	NA	86.43	31.45	NA
3	1.93	0.30	7.59	9.31	6.59	11.55	48.47	45.82	34.45
4	1.68	0.20	6.02	8.10	6.81	12.56	38.84	55.00	38.28
5	1.05	0.06	4.89	9.85	5.27	12.61	52.83	58.67	46.83
6	0.89	0.05	6.66	9.68	8.98	10.89	59.30	64.98	44.84
7	1.06	0.06	9.06	10.93	9.68	10.39	67.14	64.33	50.42
8	2.03	0.03	9.62	10.63	9.54	10.15	65.21	75.04	40.25
9	1.74	0.04	9.50	8.49	9.65	9.29	52.77	61.66	42.45
10	1.81	NA	9.77	9.85	NA	10.29	66.00	NA	50.28
11	1.87	0.03	9.13	8.02	6.37	9.84	56.33	83.98	39.33
12	1.95	0.02	9.09	8.50	5.61	10.67	73.69	92.62	40.89
13	2.12	0.02	7.44	8.30	6.26	8.93	70.03	92.51	44.97
14	1.60	NA	7.18	8.11	NA	9.65	77.08	NA	50.09
15	1.65	0.02	7.24	8.42	5.18	9.71	68.93	76.95	53.88
16	1.45	NA	7.77	7.16	NA	10.09	73.80	NA	47.42
17	1.45	0.08	7.34	7.25	5.93	10.80	61.30	61.59	52.93
18	1.71	NA	6.73	7.21	NA	9.83	64.43	NA	55.33
19	1.61	0.28	6.82	8.35	7.03	10.28	115.78	51.71	52.22
20	2.16	NA	5.95	7.75	NA	11.31	94.82	NA	49.78
21	2.71	0.01	6.64	7.56	0.01	11.66	97.16	12.43	48.09
22	3.72	NA	6.70	8.83	NA	10.56	95.12	NA	47.13
23	3.23	0.33	5.58	7.50	7.91	11.75	44.80	59.77	53.20
24	2.28	0.19	4.15	8.28	7.10	9.79	91.28	54.84	47.72
25	1.41	NA	3.15	7.96	NA	9.96	51.96	NA	50.24
26	1.70	0.14	4.30	7.05	5.84	10.51	100.64	51.29	55.88
27	1.83	NA	6.58	7.01	NA	11.42	98.01	NA	44.64
28	1.51	0.14	3.88	7.81	4.94	17.97	121.06	43.51	70.99
29	2.39	0.24	0.69	6.31	5.48	20.17	53.76	47.09	99.37
30	1.21	0.44	NA	7.34	6.89	NA	70.83	38.94	NA
31	1.26	NA	0.05	6.33	NA	22.50	59.00	NA	95.55
32	2.28	1.79	NA	7.53	6.65	NA	77.90	32.28	NA
33	1.48	2.55	0.27	6.61	8.68	21.58	76.53	31.68	79.29
34	2.25	NA	NA	7.04	NA	16.18	46.23	NA	83.78
35	2.41	2.00	0.00	8.05	8.04	21.62	71.16	32.57	77.08
36	2.15	0.93	0.25	8.13	7.50	21.87	78.19	39.35	97.11
37	1.45	NA	NA	8.14	NA	NA	50.28	NA	NA
38	2.69	0.35	0.05	6.15	7.29	21.81	61.95	41.05	96.93
39	1.76	1.15	NA	7.55	9.60	NA	66.74	52.43	NA
40	1.17	NA	NA	7.70	NA	17.90	84.41	NA	132.31
41	1.79	0.12	NA	6.18	13.09	18.99	48.75	114.23	129.85
42	2.26	NA	NA	6.63	16.36	NA	50.23	140.78	NA
43	1.53	NA	NA	8.90	NA	19.28	88.78	NA	188.24
44	1.91	0.13	NA	7.12	14.81	NA	57.62	134.31	NA
45	2.85	NA	NA	5.02	NA	NA	34.85	NA	NA
46	1.82	0.17	NA	6.61	NA	NA	69.07	NA	NA
47	2.53	NA	NA	7.43	16.67	NA	52.66	106.20	NA
48	2.49	NA	NA	5.33	NA	NA	59.85	NA	NA
49	2.52	NA	NA	6.46	15.63	NA	54.04	133.11	NA
50	2.77	0.00	NA	6.75	12.50	NA	62.57	164.66	NA
51	2.14	NA	NA	6.24	NA	NA	56.67	NA	NA
52	1.87	0.01	NA	5.26	NA	NA	59.82	NA	NA
53	2.36	NA	NA	4.07	NA	NA	39.05	NA	NA
54	2.53	NA	NA	4.16	NA	NA	39.37	NA	NA
55	2.36	NA	NA	5.97	NA	NA	81.89	NA	NA
56	1.91	NA	NA	5.98	NA	NA	44.50	NA	NA
57	2.53	NA	NA	3.25	NA	NA	24.46	NA	NA
58	3.34	NA	NA	4.61	NA	NA	33.01	NA	NA
59	3.67	NA	NA	5.66	NA	NA	29.57	NA	NA
60	5.58	NA	NA	3.99	NA	NA	25.61	NA	NA

## 4 DISCUSSION

To the best of our knowledge, this is the first prospective study to have used intraosseous microdialysis for the intraoperative and postoperative monitoring of bone free flaps. For buried bone flaps, monitoring the viability of the transferred skeletal tissue is challenging. Only a few researchers have used microdialysis to monitor bone free flaps (Laure et al., 2009; Mourouzis et al., 2007; Nielsen et al., 2011). Moreover, all of the latter studies featured catheters implanted in the surrounding muscle tissue because a reliable, well‐perfused cuff of tissue around the catheter is required. For bone flaps or composite flap, the assessment of this adjacent tissue may not be sufficient and might not accurately reflect the bone metabolism. In a study of a fibular free flap, Mourouzis et al. (2007) performed microdialysis on residual fragments of the flexor hallucis longus and concluded that microdialysis catheters cannot be inserted directly into bone. Our present results show that direct monitoring of bone tissue perfusion with microdialysis is possible. Bøgehøj et al. (2007) used microdialysis to evidence the development of ischemia in femoral heads removed from patients undergoing total hip replacement. Next, the researchers explored the phenomenon in the dead space around the catheter in the drill canal and concluded that (a) an equilibration period of 2 hr is necessary so that the measured values are representative of the bone metabolism, and (b) a reference measurement in healthy bone must be made (Bøgehøj, Emmeluth, & Overgaard, 2011). In the present study, the mean equilibration period was 5 hr and 5 min; however, the specific metabolic pattern of bone tissue must be taken into account. Indeed, flaps mainly composed of muscle and those mainly composed of fat have different metabolic patterns. Although it is known that variables involved in glycolysis change faster in muscle than in other tissues in the flap, a specific analysis in bone has yet to be performed (Röjdmark, Ungerstedt, Blomqvist, Ungerstedt, & Hedén, 2002). Our study revealed the metabolic changes in bone tissue after a period of ischemia (intraoperatively and during the first 5 days postsurgery). In a recent review of objective methods for the intraoperative assessment of free flap perfusion, Smit et al. (2018) cited only one article on microdialysis (Edsander‐Nord, Röjdmark, & Wickman, 2002) and concluded that additional studies were necessary.

Our present results demonstrate that the microdialysis reference values for soft tissues cannot be applied to bone. For example, a lactate concentration above 10 mmol/L corresponds to a Level 1 alert, and 15 mmol/L is considered to be critical in soft tissues (Birke‐Sørensen et al., 2010). The distribution of lactate values that we obtained in fibula and iliac crest free flaps does not corroborate the soft tissue alert values. Similarly, we found a mean glucose level of 2.02 mmol/L during the monitoring period; even though this is higher than the alert value described by Birke‐Sørensen et al. (2010) for muscle free flaps, it is almost three times lower than the value of 6 mmol/L considered by Setälä et al. (2006) to be the normal value in soft tissue. Forty percent of the glucose values recorded in our study were between 0 and 1 mmol/L, which is considered as a Level 1 alert in muscle free flaps (Figure 4A–C). These initial results suggest the presence of a specific metabolic pattern in bone free flaps. Our data might help to gain a better understanding of the behavior of bone tissue in chimeric flaps. However, this pilot clinical study of intraosseous microdialysis for bone free flap monitoring presented a number of limitations. Firstly, the number of patients was relatively small. Secondly, the microdialysis values in patients with flap failure were only correlated with ischemia retrospectively and thus did not enable surgical revision—even though the lactate/pyruvate ratio values accurately indicated the perfusion failure in both cases. Nonetheless, it should be borne in mind that the specific metabolic profile of bone in free flaps had not been investigated until now; this explains why the microdialysis measurements alone did not prompt us to perform flap revision surgery—especially since the flaps were buried, and there was no skin paddle to support the microdialysis data. In clinical practice, the use of microdialysis to monitor bone free flaps might ultimately allow ischemic phenomena to be detected before the first clinical signs. The technique itself has certain limitations—primarily the lack of a more secure way to anchor the catheter. Our loss rate of 11.8% might be acceptable for a feasibility study but not for routine clinical practice. Moreover, the clinical use of microdialysis will certainly generate additional costs. However, Setälä, Koskenvuori, Gudaviciene, Berg, and Mustonen (2009) considered that the extra costs of using microdialysis would be covered if one or two flaps per year were saved by more effective monitoring. Furthermore, an equilibrium period of about 5 hr seems to be required before relevant data can be collected for bone free flaps. Although this may be a disadvantage for clinical use, it corroborates the fact that bone free flaps have a particular metabolic pattern. Finally, the use of microdialysis certainly requires additional work but the staff compliance rate for sample collection of 80% in our study was more than acceptable—especially since microdialysis provides objective, quantitative values that are likely to reassure medical teams with little experience of monitoring buried flaps. In view of (a) the abovementioned factors, (b) the challenging nature of monitoring of bone free flaps, and (c) the financial and human costs of surgical revision, it seems reasonable to take advantage of the additional assistance provided by microdialysis. This is why further studies of a larger numbers of patients will now be required to define the specific alert value for each metabolite in bone free flaps in order to integrate this technique into the monitoring resources made available to surgeons in the future.

## 5 CONCLUSION

Our present results demonstrate the feasibility of intraosseous microdialysis for safely exploring bone perfusion in free flaps. This technique allows the specific assessment of bone tissue viability. Given that our prospective measurements were correlated with the clinical outcomes, microdialysis data may be of value to clinicians in the early diagnosis of ischemic events. With a view to broadening the clinical use of this technique, it is now necessary to define the specific alert value for each metabolite in bone free flaps and to specify the latter's metabolic pattern, specifically after reperfusion.
